# Supplementary material for: Self-stigma among clients of outpatient psychiatric clinics: A cross-sectional survey
Source: PLoS One. 2022 Jul 1;17(7):e0269465. doi: 10.1371/journal.pone.0269465 (PMC9249178; doi:10.1371/journal.pone.0269465)
Supplement: S1 Table — (DOCX) [file pone.0269465.s001.docx]

S1 Table. Overall p-values and also p-values from pairwise comparisons when overall p-value less than 0.05

|  | **N** | **Awareness** | **Agreement** | **Application** | **Harm to self-esteem** | **Total** |
| --- | --- | --- | --- | --- | --- | --- |
| **Education***   1. Basic 2. General/Vocational 3. Higher   **Overall p-value** | 141  501  251 | 0.066 | 0.060 | a vs b; p=0.063  a vs c; p=0.001  b vs c; p=0.030  0.004 | 0.204 | 0.204 |
| **Employment status***   1. Employed/student 2. Unemployed 3. Rehab. support/sick leave 4. Retired 5. Other   **Overall p-value** | 313  154  115  264  51 | 0.104 | 0.961 | a vs b; p=0.002  a vs c; p=0.001  a vs d; p=0.003  a vs e; p=0.237  b vs c; p=0.673  b vs d; p=0.592  b vs e; p=0.437  c vs d; p=0.342  c vs e; p=0.291  d vs e; p=0.640  0.002 | a vs b; p<0.001  a vs c; p<0.001  a vs d; p=0.072  a vs e; p=0.306  b vs c; p=0.580  b vs d; p=0.018  b vs e; p=0.144  c vs d; p=0.006  c vs e; p=0.070  d vs e; p=0.980  <0.001 | 0.055 |
| **Living situation***  a) Alone  b) With family  c) With relatives/friends  d) Supported/at half-way home  **Overall p-value** | 422  439  19  18 | a vs b; p=0.002  a vs c; p= 0.787  a vs d; p=0.234  b vs c; p=0.530  b vs d; p=0.768  c vs d; p=0.506  0.017 | 0.567 | a vs b; p=0.102  a vs c; p=0.208  a vs d; p=0.030  b vs c; p=0.426  b vs d; p=0.008  c vs d; p=0.013  0.045 | a vs b; p=0.330  a vs c; p=0.832  a vs d; p=0.003  b vs c; p=0.727  b vs d; p=0.002  c vs d; p=0.047  0.036 | 0.281 |
| **Mental disorder***  a) Affective disorder  b) Psychotic disorder  c) Other  **Overall p-value** | 636  242  17 | a vs b; p<0.001  a vs c; p= 0.003  b vs c; p=0.112  <0.001 | 0.178 | 0.258 | a vs b; p=0.001  a vs c; p=0.200  b vs c; p=0.756  0.003 | a vs b; p<0.001  a vs c; p=0.119  b vs c; p=0.748  <0.001 |
| **Mental disorder (length)***  a) Under 5 years  b) 5-15 years  c) 16-25 years  d) Over 25 years  **Overall p-value** | 218  400  176  56 | 0.372 | a vs b; p=0.990  a vs c; p= 0.045  a vs d; p=0.026  b vs c; p=0.027  b vs d; p=0.019  c vs d; p=0.400  0.020 | a vs b; p=0.026  a vs c; p=0.001  a vs d; p=0.021  b vs c; p=0.124  b vs d; p=0.279  c vs d; p=0.940  0.007 | 0.481 | a vs b; p=0.334  a vs c; p=0.011  a vs d; p=0.041  b vs c; p=0.052  b vs d; p=0.115  c vs d; p=0.774  0.031 |
| **Outpatient care (length)***  a) Under 1 year  b) 1-5 years  c) 6-10 years  d) Over 10 years  **Overall p-value** | 99  374  187  186 | 0.154 | 0.138 | a vs b; p=0.518  a vs c; p=0.071  a vs d; p=0.017  b vs c; p=0.092  b vs d; p=0.013  c vs d; p=0.477  0.024 | 0.537 | 0.291 |
| **PHQ-9 (categorized)***  a) None (0-4)  b) Mild (5-9)  c) Moderately (10-14)  d) Moderately severe (15-19)  e) Severe (20-27)  **Overall p-value** | 143  256  204  164  121 | a vs b; p<0.001  a vs c; p<0.001  a vs d; p<0.001  a vs e; p<0.001  b vs c; p=0.577  b vs d; p=0.231  b vs e; p<0.001  c vs d; p=0.010  c vs e; p<0.001  d vs e; p=0.039  <0.001 | a vs b; p=0.006  a vs c; p=0.004  a vs d; p<0.001  a vs e; p<0.001  b vs c; p=0.723  b vs d; p=0.016  b vs e; p=0.017  c vs d; p=0.048  c vs e; p=0.046  d vs e; p=0.855  <0.001 | a vs b; p<0.001  a vs c; p<0.001  a vs d; p<0.001  a vs e; p<0.001  b vs c; p<0.001  b vs d; p<0.001  b vs e; p<0.001  c vs d; p=0.015  c vs e; p<0.001  d vs e; p=0.091  <0.001 | a vs b; p<0.001  a vs c; p<0.001  a vs d; p<0.001  a vs e; p<0.001  b vs c; p<0.001  b vs d; p<0.001  b vs e; p<0.001  c vs d; p=0.026  c vs e; p<0.001  d vs e; p<0.001  <0.001 | a vs b; p<0.001  a vs c; p<0.001  a vs d; p<0.001  a vs e; p<0.001  b vs c; p<0.001  b vs d; p<0.001  b vs e; p<0.001  c vs d; p=0.007  c vs e; p<0.001  d vs e; p=0.003  <0.001 |
