# Supplementary material for: Self-stigma among clients of outpatient psychiatric clinics: A cross-sectional survey
Source: PLoS One. 2022 Jul 1;17(7):e0269465. doi: 10.1371/journal.pone.0269465 (PMC9249178; doi:10.1371/journal.pone.0269465)
Supplement: Attachment — Submitted filename: Review_self-stigma.docx [file pone.0269465.s002.docx]

The manuscript is well-written. It gives a detailed picture of self-stigma among psychiatric patients attending outpatient clinics. It highlights that the experiences of self-stigma among mentally ill people are prevalent irrespective of the living place or the country. Also, the exploration of factors associated with self-stigma is similar to previous studies, which suggests that future work should focus on this field to explore further the occurrence of self-stigma among the risk group of people.

Some suggestions mentioned below might strengthen the value of the manuscript and add more detail about the experiences of self-stigma among mentally ill patients.

Major corrections

**Rationale of the study:** There are plenty of data related to factors associated with self-stigma and its prevalence. However, the knowledge gap and the need for the study in Finland have not been addressed.

**Line 147:** **Inclusion criteria:** There is no information regarding the insight of the respondents. How did you assume that the respondents had a good insight to answer the questionnaire? Had the insight been checked before distributing the questionnaire? If yes, the detailed information should be included in the “sampling method and patient recruitment” section. If not, it should be mentioned in the study's limitations, which would greatly influence the result of this study.

As found in results, patients with affective disorder had high self-stigma. Did you analyze affective disorder and depressive symptoms?

**Population of the study:** From the beginning of the abstract, it is clear that the study population is persons with affective and psychotic disorders. However, in the result section, 17 people were categorized as others (diagnosis). Therefore, it is suggested to justify it or remove those 17 and reanalyze. It may affect the final concluding result as explained in the manuscript “affective disorder people have higher experiences of self-stigma”.

**Minor corrections**

Line 126: The meaning of statement is not clear. Please rewrite

Line 130: The statement would be difficult for people outside Finland to understand. Please specify if these 16 outpatient clinics are run by one district hospital or in one district hospital, there are 16 outpatient clinics. Maybe providing brief introduction regarding Finish healthcare would be appropriate.

**Clinical characteristics:** type of mental disorder (other) should be clarified as mentioned above

**Instruments: PHQ-9** Please indicate the language of the questionnaire used. What was the validity and reliability of this tool in Finish sample?

**Line 246:** Suggested “respondents” despite “participants” throughout the manuscript as it is a quantitative study.

**Table 1 (variables)**

Mental disorder (length): 5-15 years Hyphen is missing

Psychiatric hospital care: It appears that only 388 out of 447 reported the duration. Please move 388 to column 1 for clarity

**Table 2**

The title says N=898. However, none of the variables have N=898. It might be important to explain what was considered as a missing variable and how were those variables treated?

Line 320: Please correct the typo

**Line 322:** In this study, self-stigma is the dependent variable, but the writing looks the opposite.

**Line 325-327:** Which data table does this statement refer to?

Line 350-352 The meaning of this sentence is not clear.

Limitation: Some of the limitations are against the main rationale of this manuscript and some are not relevant. I recommend removing the first and fourth limitation

Fifth limitation needs more elaboration explaining how psychosocial factors could have impacted on experiences of self-stigma.

Last limitation could be the recommendation from this study

Conclusion: line 424-425 in irrelevant in conclusion
