# Supplementary material for: Self-stigma among clients of outpatient psychiatric clinics: A cross-sectional survey
Source: PLoS One. 2022 Jul 1;17(7):e0269465. doi: 10.1371/journal.pone.0269465 (PMC9249178; doi:10.1371/journal.pone.0269465)
Supplement: Attachment — Submitted filename: Response to Reviewers.docx [file pone.0269465.s003.docx]

Maritta Välimäki

Central South University

School of Nursing

China

PLOS ONE	10th January 2022

Stephan Doering

**Responses to the Editor’s and Reviewers’ comments**

We are thankful for the valuable comments received on our manuscript [PONE-D-21-27914] entitled “Self-stigma among clients of outpatient psychiatric clinics: A cross-sectional survey”.

We found the comments very useful in improving the quality of the manuscript, and we have revised the paper accordingly. The reviewers’ comments are numbered 1., 2., etc., and specific modifications can be found under each question. Revisions in the manuscript are highlighted in yellow. We hope that these modifications satisfy the evaluators of the manuscript.

According to the request, please correct the online submission form with the following statements in the Competing Interests section:

“The first author, Ninni Ihalainen, is affiliated with the hospital involved in the study. This does not alter our adherence to PLOS ONE policies on sharing data and materials.”

In addition, we are not allowed to make data publicly available due to legal and ethical restrictions. Therefore, please add the statements to the Data Availability section:

“The datasets underlying this study are not publicly available due to the restriction set by the Finnish Data Protection Act (Finlex 1050/2018). In addition, the ethical statement of this study (The Ethics Committee of the University of Turku [ID 55/2018]) does not contain permission to share data for any other purpose than this current study. Please contact the corresponding author, Maritta Välimäki, if further information is desired: [maritta.valimaki@csu.edu.cn](mailto:maritta.valimaki@csu.edu.cn).”

If further adjustments to the manuscript are needed, please let us know, and we will be happy to comply.

On behalf of all authors,

Maritta Välimäki, Professor

**REVIEWERS’ COMMENTS AND RESPONSES:**

**Reviewer #1**: The article reports findings of cross-sectional survey investigating self-stigma among a convenience sample of outpatients in Finland. The topic is generally interesting, however I identified several issues that should be adressed before publication:

**1)** In the introduction the authors state that the topic was already investigated in many studies, which had conflicting findings. I agree that there is a need for additional investigations, however in a first step the reasons for the inconsistent findings of past literature should be identified and adressed using more rigorous methods (e.g. longitudinal designs). I am not sure how another cross-sectional study (using a convenience sample) can help to better understand self-stigma among people with mental illness. What new information is provided?

**RESPONSE**: We clarified the purpose of the study and added justifications as follows: “Despite the many existing studies, knowledge about factors related to self-stigma are still uncertain and partially contradictory. First, most studies in this area target persons with schizophrenia, and less is known about self-stigma in relation to other mental disorders [2, 3, 19, 20, 22]. Second, in most previous studies, the sample size of the participants has been small, typically between 80 and 280. To better generalize the study results, characteristics of self-stigmatized persons with mental disorders should be further explored with larger sample sizes [3, 12, 19, 20, 24]. Third, the interest of self-stigma has usually focused on persons with depression [15]. However, knowledge about the association between depressive symptoms and self-stigma in other mental disorders is unrepresentative [3, 11, 20]. Further, there is a lack of studies about self-stigma among persons with mental health problems in Finland, in both hospital and community settings [26]. The findings of this study would be important for policy makers to consider; after a broad mental health reform in Finland in the 1990s, most persons with mental health problems have been, and continue to be, treated in outpatient clinics [26].

To better understand self-stigma among persons living in a community, we administered a survey related to self-stigma among persons with affective disorders and psychotic disorders to determine the association of self-stigma with sociodemographic and clinical factors. The study results can form a groundwork for further large-scale studies related to stigma in persons with mental health problems in Finland.”

**2)** The authors should start the introduction with clear definitions of used concepts (i.e. self-stigma, public stigma, personal stigma,...)

**RESPONSE:** This section has been modified to make it clearer as follows:” Self-stigma, also called internalized stigma, is defined as personal internalization of the prejudice leading to self-discrimination [5]. It is also seen as a process in which a person internalizes the prejudice attitudes leading to self-discrimination, recognizes being part of a stigmatized group and becomes aware of the public stigma, agrees with stigma, applies the stigma to themselves, and then experiences harm to their self-esteem because of the stigma [6].”

**3)** I suggest to exchange Ref 10 -> the cited article is theoretical and since its publication in 2014 several experimental studies investigated this topic (partly using longitudinal data) which allow to make the statement of an association between self-stigma and suicidality.

**RESPONSE**: We removed this reference and added a more appropriate one. (Oexle, N., Rüsch, N., Viering, S., Wyss, C., Seifritz, E., Xu, Z., et.al. Self‑stigma and suicidality: a longitudinal study. Eur Arch Psychiatry Clin Neurosci. 2017;267: 359–361.

<https://doi.org/10.1007/s00406-016-0698-1>)

**4)** The authors state their data is available within the manuscript and its supporting infromation files. However, the manuscript does not include raw data (it merely reports means etc.) and no other files were available to me.

**RESPONSE:** Based on the ethical statement of this study (The Ethics Committee of the University of Turku [ID 55/2018]) and the Finnish Data Protection Act (Finlex 1050/2018) we are not allowed to make data publicly available.

According to instructions of the journal, we have requested a correction for this in a cover letter.

In the end of the submitted article more detailed statistical results (pairwise comparisons) from Table 3 are presented in supporting information S1 Table.

**Reviewer #2:** The manuscript is well-written. It gives a detailed picture of self-stigma among psychiatric patients attending outpatient clinics. It highlights that the experiences of self-stigma among mentally ill people are prevalent irrespective of the living place or the country. Also, the exploration of factors associated with self-stigma is similar to previous studies, which suggests that future work should focus on this field to explore further the occurrence of self-stigma among the risk group of people. Some suggestions mentioned below might strengthen the value of the manuscript and add more detail about the experiences of self-stigma among mentally ill patients.

**RESPONSE**: The notion of further study needed have been added into the Discussion section.

**Major corrections**

**1)** Rationale of the study: There are plenty of data related to factors associated with self-stigma and its prevalence. However, the knowledge gap and the need for the study in Finland have not been addressed.

**RESPONSE:** We clarified the knowledge gap and added justifications as follows; “ Despite the many existing studies, knowledge about factors related to self-stigma are still uncertain and partially contradictory. First, most studies in this area target persons with schizophrenia, and less is known about self-stigma in relation to other mental disorders [2, 3, 19, 20, 22]. Second, in most previous studies, the sample size of the participants has been small, typically between 80 and 280. To better generalize the study results, characteristics of self-stigmatized persons with mental disorders should be further explored with larger sample sizes [3, 12, 19, 20, 24]. Third, the interest of self-stigma has usually focused on persons with depression [15]. However, knowledge about the association between depressive symptoms and self-stigma in other mental disorders is unrepresentative [3, 11, 20]. Further, there is a lack of studies about self-stigma among persons with mental health problems in Finland, in both hospital and community settings [26]. The findings of this study would be important for policy makers to consider; after a broad mental health reform in Finland in the 1990s, most persons with mental health problems have been, and continue to be, treated in outpatient clinics [26].

To better understand self-stigma among persons living in a community, we administered a survey related to self-stigma among persons with affective disorders and psychotic disorders to determine the association of self-stigma with sociodemographic and clinical factors. The study results can form a groundwork for further large-scale studies related to stigma in persons with mental health problems in Finland.”

**2)** Line 147: Inclusion criteria: There is no information regarding the insight of the respondents. How did you assume that the respondents had a good insight to answer the questionnaire? Had the insight been checked before distributing the questionnaire? If yes, the detailed information should be included in the “sampling method and patient recruitment” section. If not, it should be mentioned in the study's limitations, which would greatly influence the result of this study.

**RESPONSE:** We have clarified this information in the “sampling method and patient recruitment” section as follows: “No formal capacity test was implemented to assess patients’ insight; rather, we relied on the judgment of the health care professionals who met with the patients regularly.”

We have also taken this into account in the section of limitations as follows; “the data collection was based on respondents’ self-reported information, so, precise information about diagnoses, treatment history and other objective measures could not be collected. We may therefore question the validity of the respondents’ own categorization of their diagnostic group. We can also speculate whether participants even had the insight to identify themselves in specific diagnosis category. For example, 17 patients responded with “Other” if they felt that they did not belong to either diagnostic group or if they were unsure to which diagnostic group they belonged. Indeed, it has been shown that some persons with mental illness may not be aware of their own diagnosis or that they lack insight on their illness. We did not use a specific assessment method to assure respondents’ mental status or insight for screening purposes during patient recruitment. It would have been helpful to have been able to confirm patients’ capacity or insight; not having been able to caused a potential point of risk. On the other hand, the service system in the study organization is divided into different pathways based on diagnostic groups: one for those with affective disorders and one for those with psychotic disorders. Respondents’ categorization of their diagnoses could then be assured by comparing organization records. “

**3)** As found in results, patients with affective disorder had high self-stigma. Did you analyze affective disorder and depressive symptoms?

**RESPONSE:** We study association between three types of mental disorder and depression as classified into five categories with Fisher's exact test and we found strong association (p<0.0001). In affective disorder group, subjects had clearly more severe depression symptoms than in other two groups.

**4)** Population of the study: From the beginning of the abstract, it is clear that the study population is persons with affective and psychotic disorders. However, in the result section, 17 people were categorized as others (diagnosis). Therefore, it is suggested to justify it or remove those 17 and reanalyze. It may affect the final concluding result as explained in the manuscript “affective disorder people have higher experiences of self-stigma”.

**RESPONSE:** 17 ‘other’ subjects were included in our analyses. We feel that they have minor impact to the results, but it would be important to include all respondents to the analyses. However, if reviewers strongly feels that we should remove those subjects, we are ready to do it.

**Minor corrections**

**5)** Line 126: The meaning of statement is not clear. Please rewrite.

**RESPONSE:** We modified this statement and added new reference as follows:” The design was suitable for our purposes as we examined the phenomenon of self-stigma and relationships among self-stigma at a fixed point in time [27].“

**6)** Line 130: The statement would be difficult for people outside Finland to understand. Please specify if these 16 outpatient clinics are run by one district hospital or in one district hospital, there are 16 outpatient clinics. Maybe providing brief introduction regarding Finish healthcare would be appropriate.

**RESPONSE**: This Setting section has been modified to make it more informative as follows; “The study was conducted at 16 outpatient clinics that are part of one hospital district in southern Finland. The hospital district currently serves 479,341 inhabitants in its area [28]. The specific hospital district was selected as it represents a typical Finnish hospital district including specialized psychiatric care. The outpatient clinics were selected as they offer specialized psychiatric treatment focused on affective disorders or psychotic disorders. In general, primary health care services are responsible for mental health care. In more serious cases, clients are treated in the outpatient clinics or psychiatric wards for specialized health care with a referral by a medical doctor at a primary health care center or in private health care services [29]. In outpatient clinics, clients have monthly appointments with their contact person, typically with a psychiatric nurse. Appointments include interactive discussions with therapeutic and supportive approaches or group activities such as with functional and peer groups.“

**7)** Clinical characteristics: type of mental disorder (other) should be clarified as mentioned above

**RESPONSE:** We clarified ”Other” in the sections of methods as follows; “Clinical characteristics: a type of mental disorder (affective disorder, psychotic disorder or other, if a respondent felt that their mental disorder could not be categorized as affective or psychotic).

We clarified this in Limitations also.

**8)** Instruments: PHQ-9 Please indicate the language of the questionnaire used.

**RESPONSE**: We added the sentence as follows: ” For this study, the Finnish language version of the PHQ-9 instrument, available online, was used.”

**9)** What was the validity and reliability of this tool in Finish sample?

**RESPONSE:** We added the values of the Cronbach’s alpha for each variable (ranged from 0.85 to 0.87), and for the total scale value (0.88) in the Instruments section.

**10)** Line 246: Suggested “respondents” despite “participants” throughout the manuscript as it is a quantitative study.

**RESPONSE:** Throughout the manuscript ”participants” has been removed and ”respondents” has been added instead.

Table 1 (variables)

**11)** Mental disorder (length): 5-15 years Hyphen is missing

**RESPONSE:** A hyphen has been added to the table.

**12)** Psychiatric hospital care: It appears that only 388 out of 447 reported the duration. Please move 388 to column 1 for clarity

**RESPONSE:** The number 388 has been moved to column 1 in table 1.

**Table 2**

**13)** The title says N=898. However, none of the variables have N=898. It might be important to explain what was considered as a missing variable and how were those variables treated? **RESPONSE:** We did not allow any missing values when calculating the total or subscales. Therefore, N is less than 898 in every scale. We remove the "(n=898)" from the title. In addition, we added this description to the statistical methods section.

**14)** Line 320: Please correct the typo

**RESPONSE:** We corrected the sentence.

**15)** Line 322: In this study, self-stigma is the dependent variable, but the writing looks the opposite.

**RESPONSE:** We corrected the sentence as follows;” If respondents had higher mean values with depressive symptoms, they experienced a higher level of self-stigma”.

**16)** Line 325-327: Which data table does this statement refer to?

**RESPONSE:** For this statement there was no separate table, so this result is described in the text only.

**17)** Line 350-352 The meaning of this sentence is not clear.

**RESPONSE:** We clarified the sentence as follows;” Therefore, we did continue to examine this topic and found that the diagnosis of affective disorder was associated with higher scores of self-stigma than the psychotic disorder was.”

**18)** Limitation: Some of the limitations are against the main rationale of this manuscript and some are not relevant. I recommend removing the first and fourth limitation. Fifth limitation needs more elaboration explaining how psychosocial factors could have impacted on experiences of self-stigma. Last limitation could be the recommendation from this study.

**RESPONSE:** We modified the limitations section as follows;

**“**Our study has several limitations. First, the data collection was based on respondents’ self-reported information, so, precise information about diagnoses, treatment history and other objective measures could not be collected. We may therefore question the validity of the respondents’ own categorization of their diagnostic group. We can also speculate whether participants even had the insight to identify themselves in specific diagnosis category. For example, 17 patients responded with “Other” if they felt that they did not belong to either diagnostic group or if they were unsure to which diagnostic group they belonged. Indeed, it has been shown that some persons with mental illness may not be aware of their own diagnosis or that they lack insight on their illness. We did not use a specific assessment method to assure respondents’ mental status or insight for screening purposes during patient recruitment. It would have been helpful to have been able to confirm patients’ capacity or insight; not having been able to caused a potential point of risk. On the other hand, the service system in the study organization is divided into different pathways based on diagnostic groups: one for those with affective disorders and one for those with psychotic disorders. Respondents’ categorization of their diagnoses could then be assured by comparing organization records.

Second, as the study was cross-sectional, results could only be captured at specific time points. To detect any causal or long-term effects of self-stigma in persons with mental disorders, further studies are needed to answer these questions. Third, this study focused participants’ socio-demographic and clinical factors, and lacked an investigation into the association between self-stigma and any psychosocial factors as been recommended by other investigators [11, 18]. To offer a deeper insight into this complex phenomenon, factors related to self-stigma, different career pathways, life stages and psychological factors, such as hope, self-esteem, empowerment and quality of life, should be studied in the future.

Taken all together, despite the limitations, the findings from this study expand current knowledge about self-stigma and its related factors among patients living and treated in a community setting. A more context-oriented research approach with a longitudinal design could bring new understanding to how stigmatized attitudes develop as part of social integration and individual functioning and in specific contexts. Future work should also focus on further exploring the occurrence of self-stigma among vulnerable risk groups that are often excluded in society.”

**19)** Conclusion: line 424-425 in irrelevant in conclusion

**RESPONSE:** Sentence was removed.
