# Supplementary material for: Self-stigma among clients of outpatient psychiatric clinics: A cross-sectional survey
Source: PLoS One. 2022 Jul 1;17(7):e0269465. doi: 10.1371/journal.pone.0269465 (PMC9249178; doi:10.1371/journal.pone.0269465)
Supplement: Attachment — Submitted filename: comments.docx [file pone.0269465.s004.docx]

Title: Self-stigma among clients of outpatient psychiatric clinics: A cross-sectional survey

Comments to author(s)

It is meaningful to study Finnish self-stigma with a large sample size. Through the revision of the manuscript, logical and persuasive power were strengthened. I would like to make a strong suggestion in the analysis section to increase the power of results. Analysis of differences in self-stigma scores according to the characteristics of current participants provides only little information to the reader. Regression models should be built and verified through correlation analysis and multivariate regression analysis to analyze factors affecting self-stigma using such a large number of samples.

Pleased, designate the dependent variable as self-stigma and show the model fit of explanatory power when the personal factors and clinical factors (eg. Depression) are used as independent variables. The author(s) need to perform advanced statistics with the help of a statistician to further solidify their results.
