# Supplementary material for: Self-stigma among clients of outpatient psychiatric clinics: A cross-sectional survey
Source: PLoS One. 2022 Jul 1;17(7):e0269465. doi: 10.1371/journal.pone.0269465 (PMC9249178; doi:10.1371/journal.pone.0269465)
Supplement: Attachment — Submitted filename: 1Response to Reviewers27042022.docx [file pone.0269465.s005.docx]

Maritta Välimäki

Central South University

School of Nursing

China

PLOS ONE	27th April 2022

Stephan Doering

**Responses to the Reviewers’ comments**

We are thankful for the valuable comments received on our manuscript [PONE-D-21-27914R1] entitled “Self-stigma among clients of outpatient psychiatric clinics: A cross-sectional survey”.

We found the comments useful in improving the quality of the manuscript, and we have revised the paper accordingly. We hope that these modifications satisfy the evaluators of the manuscript.

On behalf of all authors,

Maritta Välimäki, Professor

**REVIEWERS’ COMMENTS AND RESPONSES:**

**Reviewer #2**: The authors have addressed the queries raised for the betterment of the manuscript. All the comments have been addressed well. One suggestion regarding type of mental disorder (affective, psychotic and others), please mention in patient recruitment section that few (2%) of the respondents had other than the affective and psychotic disorders.

**RESPONSE:** Thank you for your valuable comment, which is related to the generalizability of the results. We added the sentence to the Discussion section as follows.” In addition, we had only 2% of patients who were diagnosed with other diagnoses than affective disorder or psychotic disorder. Therefore, our study results might be generalized in these two specific study groups only. “

**Reviewer #3**: Comments to author(s)
It is meaningful to study Finnish self-stigma with a large sample size. Through the revision of the manuscript, logical and persuasive power were strengthened. I would like to make a strong suggestion in the analysis section to increase the power of results. Analysis of differences in self-stigma scores according to the characteristics of current participants provides only little information to the reader. Regression models should be built and verified through correlation analysis and multivariate regression analysis to analyze factors affecting self-stigma using such a large number of samples.
Pleased, designate the dependent variable as self-stigma and show the model fit of explanatory power when the personal factors and clinical factors (eg. Depression) are used as independent variables. The author(s) need to perform advanced statistics with the help of a statistician to further solidify their results.

**RESPONSE:** Thank you for the comment for multivariable modelling. Actually we did perform multivariable modelling for total self-stigma, but unfortunately we forget to document that in statistical methods section. However, we conducted now multivariable modelling for total and subscales. Naturally there was some associations between the explanatory variables and therefore some explanatory variables were dropped from the final models (we first include all significant factors from univariate approach and then removed non-significant one by one).  We added table 4 to illustrate multivariable models, and table 5 to show strong association between PHQ-9 and mental disorder categories.

In the text we added the multi-way analysis of variance (ANOVA) in abstract and corrected the statistical section as follows: “Modelling was continued with multi-way ANOVA including all significant factors from univariate approach. Then non-significant terms were removed from the model one by one “. We clarified the results section for multivariable approach as follows: “Further, when the final model in multivariable approach was conducted, we found that length of mental disorder (p = 0.018) and PHQ-9 (categorized) (p < 0.001) were significantly associated to self-stigma. This means that the longer the respondent had had the mental disorder and the more severe their depressive symptoms were, the higher the total score of self-stigma was (Table 4). Similarly, multivariable models were programmed to self-stigma subscales, and the results were quite in line with univariate approach. The greatest exception was that type of mental disorder dropped from two final models due strong association with PHQ-9 (Table 5).”

We have also taken this into account in the section of Discussion with sentence as follows: “While severity of the depressive symptoms and PHQ-9 were strongly associated so that in affective disorder the severity was more severe, PHQ-9 seemed to be stronger factor when total self-stigma was modelled.”

In addition, reference (25) has been updated to year 2021.
